# Transcriptome Profiling in Swine Macrophages Infected with African Swine Fever Virus (ASFV) Uncovers the Complex and Close Relationship with Host

**DOI:** 10.3390/pathogens11121411

**Published:** 2022-11-24

**Authors:** Zhaoyao Li, Wenxian Chen, Xiaowen Li, Keke Wu, Xinyan Wang, Weijun Wang, Yuwan Li, Lin Yi, Mingqiu Zhao, Hongxing Ding, Shuangqi Fan, Jinding Chen

**Affiliations:** 1 College of Veterinary Medicine, South China Agricultural University, Guangzhou 510642, China; 2 Guangdong Laboratory for Lingnan Modern Agriculture, Guangzhou 510642, China; 3 Key Laboratory of Zoonosis Prevention and Control of Guangdong Province, Guangzhou 510642, China

**Keywords:** African swine fever virus (ASFV), RNA-seq, innate immunity, inflammation, NF-κB signaling pathway

## Abstract

African swine fever virus (ASFV) is a pathogen to cause devastating and economically significant diseases in domestic and feral swine. ASFV mainly infects macrophages and monocytes and regulates its replication process by affecting the content of cytokines in the infected cells. There is a limited understanding of host gene expression and differential profiles before and after ASFV infection in susceptible cells. In this study, RNA-seq technology was used to analyze the transcriptomic change in PAMs infected with ASFV at different time points (0 h, 12 h, 24 h). As a result, a total of 2748, 1570, and 560 genes were enriched in group V12 h vs. MOCK, V24 h vs. MOCK, and V24 h vs. V12 h, respectively. These DEGs (differentially expressed genes) in each group were mainly concentrated in the KEGG (Kyoto Encyclopedia of Genes and Genomes) pathways related to innate immunization and inflammation, including the NF-κB signaling pathway, Toll-like receptor signaling pathway, TNF signaling pathway, IL-17 signaling pathway, cytokine-cytokine receptor interaction, and chemokine signaling pathway. Furthermore, the increased levels of IL-1β, TNF-α, IKKβ, CXCL2, and TRAF2 and decreased level of IκBα were validated through the qPCR method. These results suggested that ASFV infection can activate the NF-κB signaling pathway in the early stage. In general, this study provides a theoretical basis for further understanding the pathogenesis and immune escape mechanism of ASFV.

## 1. Introduction

African swine fever (ASF), a devastating disease for the livestock industry, seriously threatens global pork production and food security. African swine fever virus (ASFV) causes a virulent, hemorrhagic disease in infected domestic and wild pigs with a mortality rate of up to 100% [1]. Infected pigs show signs of high fever, systemic hemorrhage, vomiting, blood in stools, as well as respiratory disturbances. Splenomegaly and lymphatic hemorrhage could also be observed in diseased pigs. ASF was first reported in East Africa in the early 1900s, then spread to different countries in Africa, Europe, America, and Asia [2,3]. In August 2018, ASF entered China causing great economic losses [4]. Ma et al. collected and analyzed the related data about ASF cases throughout the entire China mainland and discovered that ASF transmission showed a northeast-southwest directional trend [4]. It was considered necessary to restrict the transportation of live pigs and pork products from ASF-affected areas and reduce the pig breeding density [4]. The pig industry in China contracts for almost half of global pork production; therefore, the outbreak of the ASF epidemic in August 2018 has had a great impact on the Chinese economy. It was estimated that China experienced direct economic losses of US $141 billion in September 2019 [5]. In addition, the rise in pork prices has had a negative impact on consumers. According to the OIE (2022), ASF has been reported in 35 countries since January 2020, involving more than 1,100,000 pigs with more than 1,800,000 animal losses [6]. The total consumption of animal feeds such as soy has also been affected by the declining number of pigs [5].

ASFV has a sizeable linear dsDNA genome of 170 to 193 kb, containing 150–167 open reading frames (ORF) [7]. The viral genome encodes more than 170 proteins, many of which have been confirmed to help viruses escape the host immune system by utilizing different mechanisms, including interferon (IFN) response inhibition, inflammatory response, apoptosis, and autophagy. Several members of the multigene family 360 (MGF360) and MGF505 strongly inhibited IL-1β maturation and IFN-β promoter activation in porcine alveolar macrophages (PAMs) [8]. Anyway, I239L, A276R, DP96R, and E120R were identified as having an essential role in the negative regulation of type I interferon [9,10,11,12]. Some ASFV proteins, such as A179Lp, A224Lp, and EP402R, regulate programmed cell death pathways in the early stage of viral infection. ASFV E199L protein induced a complete autophagy process in Vero and HEK-293T cells [13]. In addition, ASFV A137R protein inhibited IFN-β production through the autophagy-mediated lysosomal degradation of TANK-binding kinase 1 (TBK1) [14]. ASFV mainly infects macrophages and monocytes and regulates its replication process by affecting the content of cytokines in the infected cells. Although the immune functions of many ASFV proteins have been characterized, the mechanism by which ASFV interacts with the host remains unclear, given its large and complex genome structure.

In this study, as the primary target cells for ASFV, PAMs were used to construct a cell model of ASFV infection. RNA sequencing (RNA-seq) of the transcriptome is now a common method to analyze the gene expression difference in cells or tissues [15]. RNA-seq technology was utilized to analyze the gene expression patterns in PAMs infected with ASFV at different time points (0 h, 12 h, 24 h). This study aimed to understand the ASFV pathogenic characteristics from the perspective of immune pathway changes in PAMs before and after infection to provide a theoretical basis for further study of the pathogenesis and immune escape mechanism of ASFV.

## 2. Materials and Methods

### 2.1. Cell Culture and ASFV Infection of PAMs

The isolated PAMs were maintained in RPMI 1640 medium supplemented with 10% FBS at 37 °C with 5% CO_2_. When the number of viable cells in the T75 cell culture flask reached 7 × 10^6^, they were infected with ASFV pig/HLJ/2018 strain (MOI = 3) and incubated for 1 h at 37 °C before replacing the cell culture medium. PAMs infected for different time points (0 h, 12 h, and 24 h) were collected and used for RNA/DNA extraction.

### 2.2. Real-Time Quantitative PCR (qPCR)

Total RNA and ASFV genomic DNA were extracted from PAMs using Trizol reagent and E.Z.N.A. Viral DNA kit (OMEGA, New York, NY, USA), respectively. Genomic RNA was reverse transcribed with the PrimeScript RT kit (TaKaRa, San Jose, CA, USA). In order to verify the viral load and the results obtained by the RNA-seq analysis in ASFV infected PAMs, qPCR was performed with HiScript II One Step qRT-PCR SYBR Green Kit (Vazyme, Nanjing, China) for target genes amplification in Bio-rad equipment using 96-well plates. qPCR amplification conditions were as follows: one cycle of 95 °C for 30 s, 40 cycles of 95 °C for 10 s and 60 °C for 30 s, and one cycle of 95 °C for 15 s, 60 °C for 1 min and 95 °C for 15 s. All samples were analyzed in triplicate. The relative mRNA levels of ASFV and host genes were normalized to the swine β-actin mRNA level. Relative expression levels of the target genes were calculated using the comparative cycle threshold (2^−ΔΔCT^) method [16]. The information about qPCR primers related to ASFV detection and other host genes is listed in Table 1.

### 2.3. Transcriptome Sequencing and Data Analysis

The total RNA of PAMs was extracted using Trizol Reagent following the manufacturer’s protocol. mRNA was enriched by magnetic beads with Oligo(dT) and fragmented by fragmentation buffer. The cDNA, which utilized the above RNA as a template, was synthesized in the presence of random hexamers, DNA polymerase I, dNTPs, and RNase H. The amplified cDNA was subjected to end repair and adaptor ligation, and PCR. The PCR products were purified by AMPure XP beads to obtain the final cDNA library. The cDNA library was sequenced using Illumina high-throughput sequencing platform and converted to Raw Reads. After quality control and filtration by fastp software and Bowtie2 software, the filtering readings were aligned against with reference genome using HISAT2, and the differential expression conditions of genes among different samples were analyzed using RSEM and edgeR. The false discovery rate (FDR) ≤ 0.05 and the criteria of a fold difference |log2FC| ≥ 1 were considered for DEGs. GO (Gene Ontology) analysis and KEGG analysis were performed using cluster Profiler software. All data were obtained from at least three replicates. Values of *p* ≤ 0.05 were assumed to be statistically significant.

### 2.4. Statistical Analysis of Alternative Splicing Events

Alternative Splicing (AS) is a common gene expression mode in most eukaryotic cells. In eukaryotes, different mature mRNA and proteins can be produced by the same gene encoding mRNA through different splicing modes. The rMATS is a software developed for RNA-seq data, which can classify not only alternative splicing events but also perform differential analysis of alternative splicing events between different samples. In this study, rMATS was used to quantify the expression of alternative splicing events in different samples.

## 3. Results

### 3.1. ASFV Infection Conditions in PAMs

**Figure 1:**
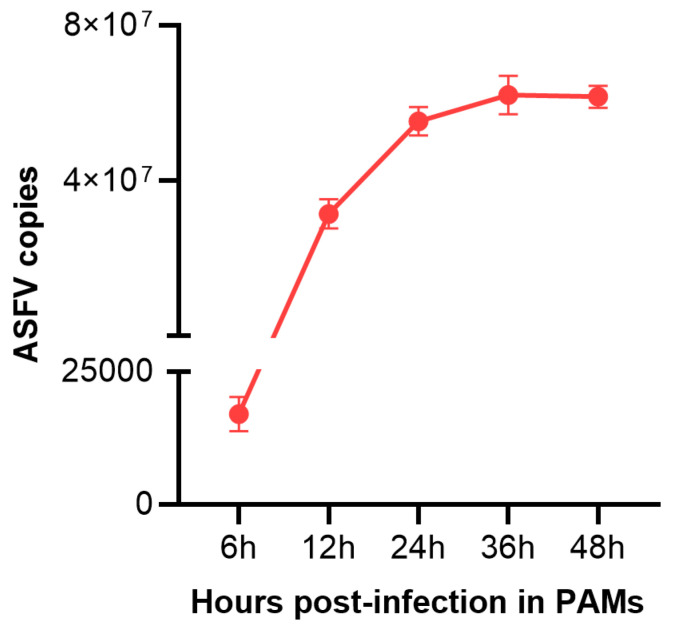
The viral copies number in PAMs infected with ASFV for different times (6 h, 12 h, 24 h,36 h, 48 h). Data are presented as mean ± SD of three independent experiments.

To validate whether PAMs were successfully infected with ASFV, the viral load in PAMs infected with ASFV (MOI = 3) for 0 h,12 h, and 24 h were determined utilizing qPCR methods. As shown in Figure 1, ASFV was detected from ASFV-infected PAMs, and the viral load in cells increased over time. The results indicated that PAMs were successfully infected with ASFV.

### 3.2. Gene Expression Statistics and Differential Analysis among Different Samples

**Figure 2:**
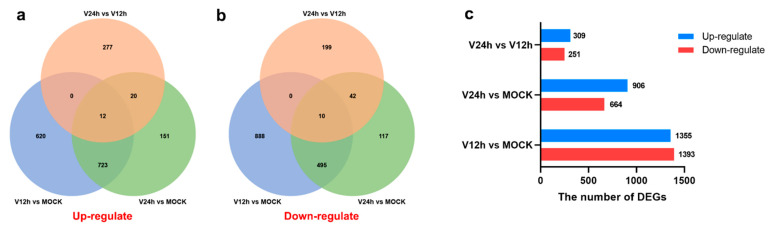
(**a**) Venn diagram of significantly upregulated genes in different groups. (**b**) Venn diagram of significantly downregulated genes in different groups. (**c**) The total number of up/down-regulated DEGs in different groups (FDR ≤ 0.05 and |log2FC| ≥ 1).

The correlation of transcriptome gene expression among infected and mock-infected samples was analyzed, and the Pearson correlation coefficient was obtained to make a heatmap. In addition, PCA (Principal Component Analysis) analysis was also performed. These results showed that the sample biological repeatability in each group as well. In addition, the V12 h and V24 h groups were significantly different from the control group. The transcriptome profiles of each group (V12 h vs. MOCK, V24 h vs. MOCK, V24 h vs. V12 h) were compared using edgeR software, and the DEGs quantities were identified based on the screening conditions of DEGs with FDR ≤ 0.05 and |log2FC| ≥ 1. In addition, compared with the V12 h group, the number of DEGs in the V24 h group was 560, among which the level of 309 genes was upregulated (Figure 2c). The results of the Venn diagram revealed that the number of common upregulated and downregulated DEGs in the three groups was 12 and 10, independently (Figure 2a,c).

**Figure 3:**
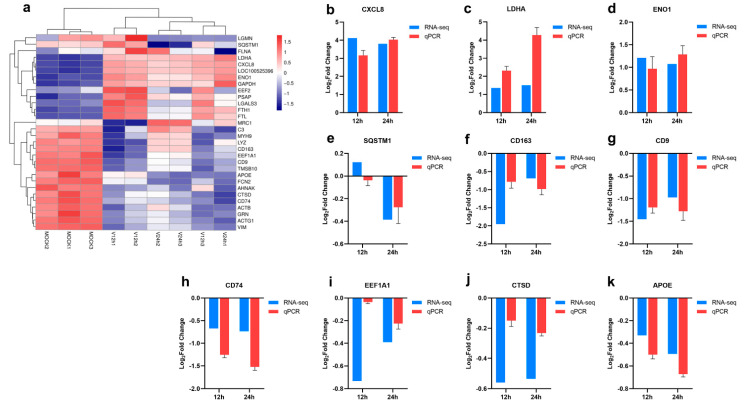
Validation of RNA-seq data by RT-qPCR. (**a**) Cluster heatmap of the top 30 expressed genes. (**b**–**k**) RT-qPCR validation of representative 10 genes selected from the top 30 expressed genes.

Hierarchical clustering analysis of the relationship between samples and genes was performed based on gene expression, and the clustering results of the top 30 expressed genes are shown in Figure 3a. Furthermore, 10 genes (CXCL8, LDHA, ENO1, SQSTM1, CD163, CD9, CD74, EEF1A1, CTSD, APOE) were picked out for RT-qPCR to verify the accuracy of the RNA-seq data. As shown in Figure 3b–k, the RT-qPCR results of these genes were generally consistent with their transcriptomic data, which indicated the accuracy and validity of the RNA-seq data.

### 3.3. KEGG Pathway Analysis of DEGs

**Figure 4:**
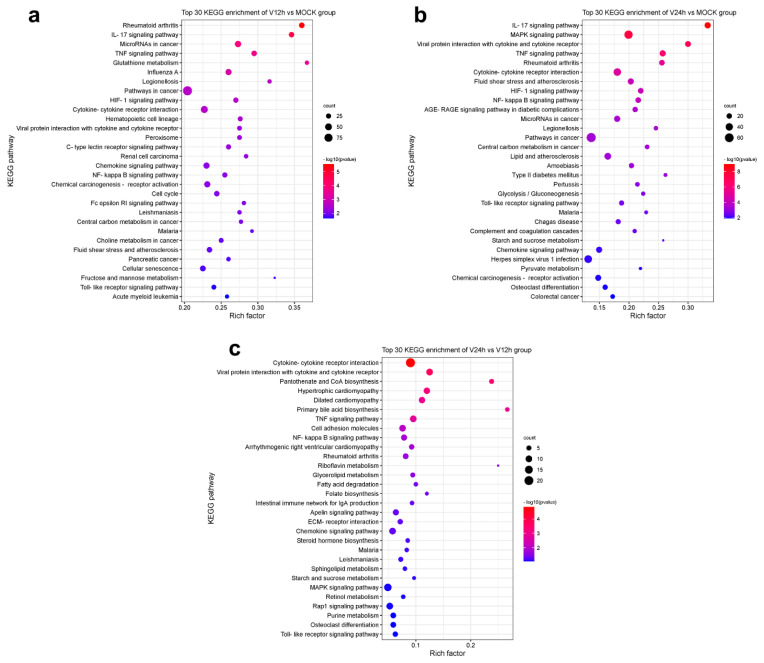
Top 30 KEGG enrichment pathways in each group. (**a**) V12 h vs. MOCK. (**b**) V24 h vs. MOCK. (**c**) V24 h vs. V12 h.

To further clarify the relevant pathways and potential biological functions involved in differentially expressed genes, the KEGG database was used for enrichment analysis of the DEGs in each group. The enrichment results of the top 30 KEGG pathways with small P-values are shown in Figure 4. Compared with the MOCK group, the DEGs in the V12h group (Figure 4a) were mainly concentrated in the KEGG pathways related to innate immunization and inflammation, including the NF-κB signaling pathway, Toll-like receptor signaling pathway, TNF signaling pathway, IL-17 signaling pathway, cytokine-cytokine receptor interaction, and chemokine signaling pathway, which were similar to the enrichment results of the other two groups. In addition, the DEGs were also involved in the metabolism regulation containing glutathione metabolism, HIF-1 signaling pathway, central carbon metabolism in cancer, choline metabolism in cancery, and fructose and mannose metabolism. The DEGs in the V24 h group (Figure 4b) compared to MOCK were also abundant in the MAPK signaling pathway, glycolysis/gluconeogenesis, starch and sucrose metabolism, and pyruvate metabolism. In addition, the DEGs of V24 h vs. V12 h (Figure 4c) were also involved in pantothenate and CoA biosynthesis, glycerolipid metabolism, fatty acid degradation, riboflavin metabolism, sphingolipid metabolism, primary bile acid biosynthesis, etc. The above results showed the profound changes in different signaling pathways and cytokines in host cells after ASFV invasion. The host cells resist virus invasion by regulating sundry innate immunity and metabolism pathways, while viruses may achieve the purpose of persistent infection by influencing host cell responses.

### 3.4. The Activation of the NF-κB Signaling Pathway in ASFV-Infected PAMs

**Figure 5:**
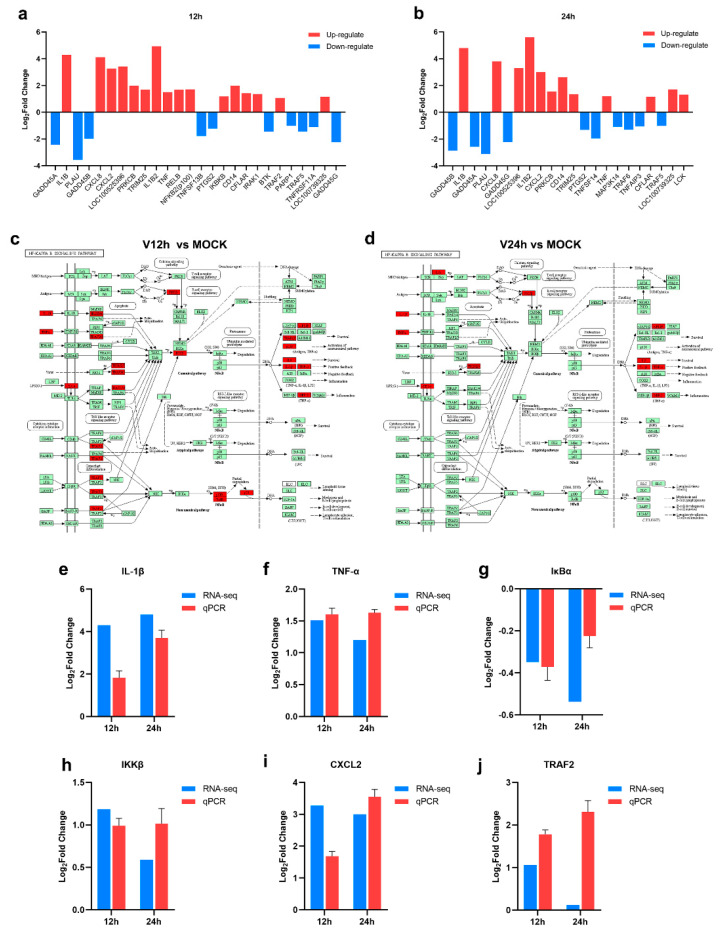
The activation of NF-κB signaling pathway in ASFV infected PAMs (**a**,**b**) The DEGs enriched in the NF-κB signaling pathway in PAMs infected with ASFV for different times ((**a**), 12 h; (**b**), 24 h). (**c**,**d**) The upregulated genes related to the NF-κB signaling pathway in PAMs infected with ASFV for 12 h (**c**)/24 h (**d**) (Upregulated DEGs were marked in red). (**e**–**j**) RT-qPCR validation of representative genes related to NF-κB signaling pathway.

KEGG pathway enrichment analysis of the transcriptomic data above indicated that DEGs in three groups were enriched in the NF-κB signaling pathway. After ASFV infection for 12 h, the transcriptional level of IL-1β, CXCL8, CXCL2, LOC100525396, PRKCB, TRIM25, IL-1β2, TNF-α, RELB, NFκB2, IKKβ, CD14, CFLAR, IRAK1, TRAF2, and LOC100739325 was increased, and the down-regulated genes including GADD45A, PLAU, GADD45B, TNFSF13B, PTGS2, BTK, PARP1, TRAF5, TNFRSF11A, GADD45G were enriched (Figure 5a). There are 12 upregulated genes (IL-1β, CXCL8, LOC100525396, IL-1β2, CXCL2, PRKCB, CD14, TRIM25, TNF-α, CFLAR, LOC100739325, LCK) and 10 down-regulated genes (GADD45B, GADD45A, PLAU, GADD45G, PTGS2, TNFSF14, MAP3K14, TRAF6, TNFAIP3, TRAF5) related to NF-κB signaling pathway in PAMs infected with ASFV for 24 h. NFκB2 (p100) and RELB associated with NF-κB noncanonical pathway were enriched in the V12 h group instead of the V24 h group compared to MOCK, which suggested that ASFV seems to induce both classical and nonclassical NF-κB pathways in the early stage, thus triggering a series of inflammatory processes in the infected host cell (Figure 5c,d). Furthermore, the increased levels of IL-1β, TNF-α, IKKβ, CXCL2, and TRAF2 and decreased level of IκBα were validated through the qPCR method (Figure 5e–j). In general, these results suggested that ASFV infection may activate the NF-κB signaling pathway.

## 4. Discussion

ASF vaccine development is difficult because of the large genome and complex structure of ASFV. Before the complete control and eradication of ASF, it is necessary to fully understand the infection and immune mechanisms and identify ASFV’s major immunogenic genes. ASFV mainly infects porcine monocytes and macrophages, and the early studies on ASFV were primarily in Vero cells. Macrophages are the primary target cells of ASFV and critical immune cells of the host [24]. Transcriptome differential analysis of ASFV-infected macrophages may help to understand the mechanism of host-pathogen interaction. Infectious progeny virus could be produced in cells after ASFV infection for 16 h. In this study, PAMs were used as an in vitro infection model, and the cell samples were harvested at 0 h, 12 h, and 24 h after ASFV infection. Furthermore, RNA-seq technology was used to analyze the transcriptional levels change of cells infected with ASFV at different time points. In the V12 h vs. MOCK group, a total of 2748 DEGs were identified, of which 1355 genes were upregulated and 1393 genes were downregulated (Figure 2c).

The RNA-seq results found that the level of the SQSTM1 gene decreased with time (Figure 3e), which was also confirmed through the qPCR method. The results suggested that ASFV may mediate the autophagy pathway. SQSTM1, also named P62, is one of the well-known autophagy proteins. In autophagy, autophagy receptor P62 binds to ubiquitinated proteins, then forms a complex with LC3-II proteins localized in the autophagic membrane, which are degraded in the autophagic lysosomes [25]. One study has described the enhancement of the autophagy process through the interaction between ASFV E199L and PYCR2 [11]. In addition, ASFV A137R and pI215L proteins were proven to inhibit type I interferon production by regulating the autophagy pathway [14,26]. However, other research indicated that ASFV A179L could interact with the Beclin-1 BH3 motif to inhibit autophagy [27,28]. So far, research on the relationship between autophagy and ASFV infection is still limited. ENO1 is a multifunctional protein involved in several biological and pathophysiological processes, including cell glycolysis, proliferation, migration, invasion, and tumorigenesis [29]. LDHA is a crucial component of glycolysis, promoting the expression of effector T cytokines [30]. It has been reported that many viruses can reprogram glucose metabolism in the host cells [31,32,33,34]. H1N1 infection can activate the glycolytic pathway of glucose metabolism to support efficient viral replication [35]. The increased levels of ENO1 and LDHA suggested the vital role of glycolysis in ASFV infection (Figure 3c,d). Similar to other viruses which could regulate glucose metabolism, ASFV may also maintain infection in host cells by reprogramming the glycolytic process. However, Xue et al. confirmed that ASFV infection did not significantly affect the glycolysis pathway, but the produced pyruvate in PAMs after ASFV infection enhanced the lactate production and TCA cycle, which further promoted ASFV replication and immune escape [36]. CXCL8 is the primary mediator of an inflammatory response, attracting neutrophils, basophils, NK cells, and T cells [37]. The gene level of CXCL8 in PAMs infected with ASFV for different time points was higher than MOCK group (Figure 3a). Some studies have reported that low-virulent ASFV strains (OURT88/3) can produce higher levels of CXCL8 and CXCL10 than virulent strains [37]. In addition, the enhanced transcriptional levels of other inflammation-related cytokines, including CCL4, CCL5, CXCL13, IL-1 and TNF-α were also described after ASFV infection [18,22,38]. It proves the close correlation between ASFV and inflammatory response. Infected host cells may clear the virus by inducing inflammation-related cytokines, and the virus may regulate the levels of these chemokines to maintain its replication. The relationship between ASFV and inflammation requires further investigation. As shown in Figure 3f–k, decreased levels of CD163, CD9, CD74, EEF1A1, CTSD, and APOE were observed in this study. Some studies demonstrated that genetically edited pigs lacking CD163 were non-permissive for PRRSV infection but could still be infected with the Georgia 2007/1 ASFV isolates [39,40]. CD163 may not be necessary for ASFV infection. CD9 is involved in cell adhesion, movement, activation, and differentiation. CD74 is an integral transmembrane molecule playing a role in the intracellular sorting of MHC class II molecules, T-cell and B-cell developments, dendritic cell (DC) motility, macrophage inflammation, and thymic selection [41]. Inactivation of eEF1A proteins leads to immunodeficiency, and neural and muscular defects and favors apoptosis [42]. CTSD is an aspartate lysosomal enzyme involved in the degradation of endocytosed and cellular proteins, apoptosis, and brain development [43]. APOE is involved in lipid metabolism and cholesterol metabolism [44]. These DEGs may play essential roles in opposing ASFV, which needs further study.

In this study, many DEGs in ASFV-infected PAMs were enriched in the pathways related to innate immunization and inflammation, including cytokine-cytokine receptor interaction, chemokine signaling pathway, Toll-like receptor signaling pathway, NF-κB signaling pathway, TNF signaling pathway and IL-17 signaling pathway (Figure 4). Different kinds of cytokines and chemokines produced by PAM cells can induce robust immune and inflammatory responses and play an important role in the host antiviral response. Some studies have shown diverse cytokines induced in ASFV-infected cells [18,22,38]. In addition, upon ASFV infection, a wide range of pro-inflammatory factors was secreted in the spleen and renal and gastrohepatic lymph nodes [45]. In vivo studies found that virulent ASFV can evade the host immune system and promote viral replication by delaying the inflammatory response in animals [46]. Nuclear factor kappa B (NF-κB) is an important family of transcription factors consisting of RelA (p65), RelB, c-Rel, p50/p105 (NF-κB1), and p52/p100 (NF-κB2), and can regulate the expression of multiple genes implicated in immunity, inflammation, stress and cell activity [47,48]. The NF-κB pathway is widely regarded as a typical pro-inflammatory signal transduction pathway, the activation of which can promote the secretion of inflammatory cytokines, chemokines, and adhesion molecules [22]. Analysis of transcriptome data from this study revealed that ASFV appeared to induce both classical and nonclassical NF-κB pathways in the early stage and inhibited them in the late stage (Figure 5c,d). NF-κB activated genes were found to be strongly upregulated in Porcine Macrophages infected with the highly virulent ASFV Georgia 2007/1 strain (GRG) [49]. ASFV infection activates the NF-κB signaling pathway, and the inhibitor of this pathway could restrain viral replication [22]. Additionally, several ASFV proteins have been confirmed that they have a strong ability to evade or subvert the antiviral innate immune response and can maintain their own replication by regulating various cytokines in the host [50,51]. The interplay between ASFV and NF-κB signaling is complex and two-sided. On the one hand, ASFV promotes early viral replication using the anti-apoptotic function of NF-κB. On the other hand, ASFV avoids NF-κB-mediated antiviral cytokine responses through different mechanisms of regulation [52]. Further studies are needed to understand how the host immune system interacts with the virus to determine cell survival or death in ASFV infection. It is worth noting that the DEGs were also enriched in metabolism pathways (Figure 4). Multiple viral infections trigger intracellular metabolic reprogramming to support viral replication or rapid cell growth. Wild-type adenovirus 5 (ADWT) could regulate glucose and glutamine metabolism to encourage viral genome replication [53,54]. Dengue virus (DENV)-induced autophagy regulates cellular lipid metabolism and releases free fatty acids conducive to efficient viral replication [55]. A large number of amino acids were discovered to be significantly upregulated in PAMs at the early stages of ASFV infection, and the aspartate and glutamate could promote ASFV replication [36]. In addition, the TCA cycle was critical for the replication of ASFV. TCA cycle may increase ATP and amino acid production to facilitate viral replication [36].

## 5. Conclusions

In summary, we compared and analyzed the DEGs of PAMs infected with ASFV at different time points (0 h, 12 h, 24 h) and found the significant enrichment and change of transcriptomic factors in diverse pathways, including immunization, inflammation, and metabolism. In addition, ASFV appeared to induce both classical and nonclassical NF-κB pathways in the early stage and inhibit them in the late stage. This study helps understand the interaction between ASFV and the host and lays a foundation for further exploration of the pathogenic mechanism of ASFV.

## Figures and Tables

**Table 1:** qPCR primers used in this study.

Primers	Sequence (5′-3′)	References or Genbank
ASFV-B646L-F	CTGCTCATGGTATCAATCTTATCGA	[17]
ASFV-B646L-R	GATACCACAAGATCRGCCGT	
CXCL8-F	AGC CCG TGT CAA CAT GAC TT	[18]
CXCL8-R	TGG AAA GGT GTG GAA TGC GT	
LDHA-F	CGTCAGCAAGAGGGAGA	[19]
LDHA-R	AAGCACTGGATTGGAAGCAACAA	
ENO1-F	AAGCCCTGGAGCTGCTGA	XM_021095279.1
ENO1-R	CGTACTTGCCCGACCTGTAGAA	
SQSTM1-F	GCTGCTCTTCCGACCCT	XM_003123639.4
SQSTM1-R	GCGATCTTATTCATTTGCTCC	
CD163-F	ATTCATCATCCTCGGACCCAT	[20]
CD163-R	CCCAGCACAACGACCACCT	
CD9-F	CCAGGATTTCTACAGGGACA	NM_214006.1
CD9-R	GCATAGTGGATGGCTTTCAG	
CD74-F	AGAGCAAGTGCAGCCGTGGAG	[21]
CD74-R	GGTACAGGAAGTAGGCGGTGGTG	
EEF1A1-F	AGTGCTAATATGCCTTGGTT	NM_001097418.2
EEF1A1-R	TTGTCAGTTGGACGAGTTG	
CTSD-F	ACGTGAAGAACGGCACCACC	NM_001037721.1
CTSD-R	GCCCAACAACGCAGAATTACA	
APOE-F	TGGGAGGAGTCCAAGTGGCA	NM_214308.1
APOE-R	GCTCCGTCAGTTCCTGGGTGA	
IL-1β-F	ACCTGGACCTTGGTTCTCTG	[22]
IL-1β-R	CATCTGCCTGATGCTCTTG	
TNF-α-F	TGGCCCAAGGACTCAGATCAT	[23]
TNF-α-R	TCGGCTTTGACATTGGCTACA	
IκBα-F	ACCAACCAGCCAGAAATCG	NM_001005150.1
IκBα-R	CACAGGCAAGGTGTAGAGGG	
IKKβ-F	AGAGGATCTTCTGCGAGTA	NM_001244129.1
IKKβ-R	CTTTGGGTGCGTAACTG	
CXCL2-F	GTGGAAACAGCAACTGCTCA	[18]
CXCL2-R	AGGGCTTGGTAGTTGTCAGG	
TRAF2-F	CCACCGCTACTGCTCCTACTGC	XM_005652719.2
TRAF2-R	CGCCTTCTTCATAAATGCCCTC	
GAPDH-F	TGGAGTCCACTGGTGTCTTCAC	[23]
GAPDH-R	TTCACGCCCATCACAAACA	

## Data Availability

The data presented in this study are available in the article.

## References

[1] Dixon L.K., Islam M., Nash R., Reis A.L. (2019). African swine fever virus evasion of host defences. Virus Res..

[2] Boklund A., Cay B., Depner K., Földi Z., Guberti V., Masiulis M., Miteva A., More S., Olsevskis E., Šatrán P. (2018). Epidemiological analyses of African swine fever in the European Union (November 2017 until November 2018). EFSA J. Eur. Food Saf. Auth..

[3] Wu K., Liu J., Wang L., Fan S., Li Z., Li Y., Yi L., Ding H., Zhao M., Chen J. (2020). Current State of Global African Swine Fever Vaccine Development under the Prevalence and Transmission of ASF in China. Vaccines.

[4] Ma J., Chen H., Gao X., Xiao J., Wang H. (2020). African swine fever emerging in China: Distribution characteristics and high-risk areas. Prev. Vet. Med..

[5] The Global Economic Impact of ASF—WOAH Bulletin. https://bulletin.woah.org/?panorama=02-2-2-2020-1-economic.

[6] ASF Distribution and the Situation in 2020–2022 (Based on INs, FURs and SMRs). https://www.woah.org/app/uploads/2022/02/asf-report6.pdf.

[7] Galindo I., Alonso C. (2017). African Swine Fever Virus: A Review. Viruses.

[8] Li J., Song J., Kang L., Huang L., Zhou S., Hu L., Zheng J., Li C., Zhang X., He X. (2021). pMGF505-7R determines pathogenicity of African swine fever virus infection by inhibiting IL-1beta and type I IFN production. PLoS Pathog..

[9] Correia S., Ventura S., Parkhouse R.M. (2013). Identification and utility of innate immune system evasion mechanisms of ASFV. Virus Res..

[10] Liu H., Zhu Z., Feng T., Ma Z., Xue Q., Wu P., Li P., Li S., Yang F., Cao W. (2021). African Swine Fever Virus E120R Protein Inhibits Interferon Beta Production by Interacting with IRF3 To Block Its Activation. J. Virol..

[11] Whittall J.T., Parkhouse R.M. (1997). Changes in swine macrophage phenotype after infection with African swine fever virus: Cytokine production and responsiveness to interferon-gamma and lipopolysaccharide. Immunology.

[12] Wang X., Wu J., Wu Y., Chen H., Zhang S., Li J., Xin T., Jia H., Hou S., Jiang Y. (2018). Inhibition of cGAS-STING-TBK1 signaling pathway by DP96R of ASFV China 2018/1. Biochem. Biophys. Res. Commun..

[13] Chen S., Zhang X., Nie Y., Li H., Chen W., Lin W., Chen F., Xie Q. (2021). African Swine Fever Virus Protein E199L Promotes Cell Autophagy through the Interaction of PYCR2. Virol. Sin..

[14] Sun M., Yu S., Ge H., Wang T., Li Y., Zhou P., Pan L., Han Y., Yang Y., Sun Y. (2022). The A137R Protein of African Swine Fever Virus Inhibits Type I Interferon Production via the Autophagy-Mediated Lysosomal Degradation of TBK1. J. Virol..

[15] Hrdlickova R., Toloue M., Tian B. (2017). RNA-Seq methods for transcriptome analysis. Wiley Interdiscip. Rev. RNA.

[16] Ju X., Li F., Li J., Wu C., Xiang G., Zhao X., Nan Y., Zhao D., Ding Q. (2021). Genome-wide transcriptomic analysis of highly virulent African swine fever virus infection reveals complex and unique virus host interaction. Vet. Microbiol..

[17] African Swine Fever—WOAH—World Organisation for Animal Health. https://www.woah.org/en/disease/african-swine-fever/.

[18] Yang B., Shen C., Zhang D., Zhang T., Shi X., Yang J., Hao Y., Zhao D., Cui H., Yuan X. (2021). Mechanism of interaction between virus and host is inferred from the changes of gene expression in macrophages infected with African swine fever virus CN/GS/2018 strain. Virol. J..

[19] Bao Z.Q., Liao T.T., Yang W.R., Wang Y., Luo H.Y., Wang X.Z. (2017). Heat stress-induced autophagy promotes lactate secretion in cultured immature boar Sertoli cells by inhibiting apoptosis and driving SLC2A3, LDHA, and SLC16A1 expression. Theriogenology.

[20] Zhu Z., Zhang X., Dong W., Wang X., He S., Zhang H., Wang X., Wei R., Chen Y., Liu X. (2020). TREM2 suppresses the proinflammatory response to facilitate PRRSV infection via PI3K/NF-kappaB signaling. PLoS Pathog..

[21] Zhang H., Liu C., Cheng S., Wang X., Li W., Charreyre C., Audonnet J.C., He Q. (2013). Porcine CD74 is involved in the inflammatory response activated by nuclear factor kappa B during porcine circovirus type 2 (PCV-2) infection. Arch. Virol..

[22] Gao Q., Yang Y., Feng Y., Quan W., Luo Y., Wang H., Zheng J., Chen X., Huang Z., Chen X. (2022). Effects of the NF-kappaB Signaling Pathway Inhibitor BAY11-7082 in the Replication of ASFV. Viruses.

[23] Zhu E., Wu H., Chen W., Qin Y., Liu J., Fan S., Ma S., Wu K., Mao Q., Luo C. (2021). Classical swine fever virus employs the PERK- and IRE1-dependent autophagy for viral replication in cultured cells. Virulence.

[24] Gomez-Villamandos J.C., Bautista M.J., Sanchez-Cordon P.J., Carrasco L. (2013). Pathology of African swine fever: The role of monocyte-macrophage. Virus Res..

[25] Chen C., Gao H., Su X. (2021). Autophagy-related signaling pathways are involved in cancer (Review). Exp. Ther. Med..

[26] Li L., Fu J., Li J., Guo S., Chen Q., Zhang Y., Liu Z., Tan C., Chen H., Wang X. (2022). African Swine Fever Virus pI215L Inhibits Type I Interferon Signaling by Targeting Interferon Regulatory Factor 9 for Autophagic Degradation. J. Virol..

[27] Hernaez B., Cabezas M., Munoz-Moreno R., Galindo I., Cuesta-Geijo M.A., Alonso C. (2013). A179L, a new viral Bcl2 homolog targeting Beclin 1 autophagy related protein. Curr. Mol. Med..

[28] Banjara S., Shimmon G.L., Dixon L.K., Netherton C.L., Hinds M.G., Kvansakul M. (2019). Crystal Structure of African Swine Fever Virus A179L with the Autophagy Regulator Beclin. Viruses.

[29] Fu Q.F., Liu Y., Fan Y., Hua S.N., Qu H.Y., Dong S.W., Li R.L., Zhao M.Y., Zhen Y., Yu X.L. (2015). Alpha-enolase promotes cell glycolysis, growth, migration, and invasion in non-small cell lung cancer through FAK-mediated PI3K/AKT pathway. J. Hematol. Oncol..

[30] Xu K., Yin N., Peng M., Stamatiades E.G., Shyu A., Li P., Zhang X., Do M.H., Wang Z., Capistrano K.J. (2021). Glycolysis fuels phosphoinositide 3-kinase signaling to bolster T cell immunity. Science.

[31] Zhao Y., Chahar H.S., Komaravelli N., Dossumbekova A., Casola A. (2019). Human metapneumovirus infection of airway epithelial cells is associated with changes in core metabolic pathways. Virology.

[32] Fontaine K.A., Sanchez E.L., Camarda R., Lagunoff M. (2015). Dengue virus induces and requires glycolysis for optimal replication. J. Virol..

[33] Passalacqua K.D., Lu J., Goodfellow I., Kolawole A.O., Arche J.R., Maddox R.J., Carnahan K.E., O’Riordan M., Wobus C.E. (2019). Glycolysis Is an Intrinsic Factor for Optimal Replication of a Norovirus. mbio.

[34] Fan S., Wu K., Zhao M., Yuan J., Ma S., Zhu E., Chen Y., Ding H., Yi L., Chen J. (2021). LDHB inhibition induces mitophagy and facilitates the progression of CSFV infection. Autophagy.

[35] Ren L., Zhang W., Zhang J., Zhang J., Zhang H., Zhu Y., Meng X., Yi Z., Wang R. (2021). Influenza A Virus (H1N1) Infection Induces Glycolysis to Facilitate Viral Replication. Virol. Sin..

[36] Xue Q., Liu H., Zhu Z., Yang F., Song Y., Li Z., Xue Z., Cao W., Liu X., Zheng H. (2022). African Swine Fever Virus Regulates Host Energy and Amino Acid Metabolism To Promote Viral Replication. J. Virol..

[37] Fishbourne E., Abrams C.C., Takamatsu H.H., Dixon L.K. (2013). Modulation of chemokine and chemokine receptor expression following infection of porcine macrophages with African swine fever virus. Vet. Microbiol..

[38] Sun H., Niu Q., Yang J., Zhao Y., Tian Z., Fan J., Zhang Z., Wang Y., Geng S., Zhang Y. (2021). Transcriptome Profiling Reveals Features of Immune Response and Metabolism of Acutely Infected, Dead and Asymptomatic Infection of African Swine Fever Virus in Pigs. Front. Immunol..

[39] Whitworth K.M., Rowland R.R., Ewen C.L., Trible B.R., Kerrigan M.A., Cino-Ozuna A.G., Samuel M.S., Lightner J.E., McLaren D.G., Mileham A.J. (2016). Gene-edited pigs are protected from porcine reproductive and respiratory syndrome virus. Nat. Biotechnol..

[40] Popescu L., Gaudreault N.N., Whitworth K.M., Murgia M.V., Nietfeld J.C., Mileham A., Samuel M., Wells K.D., Prather R.S., Rowland R. (2017). Genetically edited pigs lacking CD163 show no resistance following infection with the African swine fever virus isolate, Georgia 2007/1. Virology.

[41] Su H., Na N., Zhang X., Zhao Y. (2017). The biological function and significance of CD74 in immune diseases. Inflamm. Res..

[42] Abbas W., Kumar A., Herbein G. (2015). The eEF1A Proteins: At the Crossroads of Oncogenesis, Apoptosis, and Viral Infections. Front. Oncol..

[43] Payton A., van den Boogerd E., Davidson Y., Gibbons L., Ollier W., Rabbitt P., Worthington J., Horan M., Pendleton N. (2006). Influence and interactions of cathepsin D, HLA-DRB1 and APOE on cognitive abilities in an older non-demented population. Genes Brain Behav..

[44] Huang Y., Mahley R.W. (2014). Apolipoprotein E: Structure and function in lipid metabolism, neurobiology, and Alzheimer’s diseases. Neurobiol. Dis..

[45] Salguero F.J., Ruiz-Villamor E., Bautista M.J., Sanchez-Cordon P.J., Carrasco L., Gomez-Villamandos J.C. (2002). Changes in macrophages in spleen and lymph nodes during acute African swine fever: Expression of cytokines. Vet. Immunol. Immunopathol..

[46] Wang S., Zhang J., Zhang Y., Yang J., Wang L., Qi Y., Han X., Zhou X., Miao F., Chen T. (2020). Cytokine Storm in Domestic Pigs Induced by Infection of Virulent African Swine Fever Virus. Front. Vet. Sci..

[47] Dorrington M.G., Fraser I. (2019). NF-kappaB Signaling in Macrophages: Dynamics, Crosstalk, and Signal Integration. Front. Immunol..

[48] Zhang Q., Lenardo M.J., Baltimore D. (2017). 30 Years of NF-kappaB: A Blossoming of Relevance to Human Pathobiology. Cell.

[49] Cackett G., Portugal R., Matelska D., Dixon L., Werner F. (2022). African Swine Fever Virus and Host Response: Transcriptome Profiling of the Georgia 2007/1 Strain and Porcine Macrophages. J. Virol..

[50] Wang Q., Zhou L., Wang J., Su D., Li D., Du Y., Yang G., Zhang G., Chu B. (2022). African Swine Fever Virus K205R Induces ER Stress and Consequently Activates Autophagy and the NF-kappaB Signaling Pathway. Viruses.

[51] Powell P.P., Dixon L.K., Parkhouse R.M. (1996). An IkappaB homolog encoded by African swine fever virus provides a novel mechanism for downregulation of proinflammatory cytokine responses in host macrophages. J. Virol..

[52] Ayanwale A., Trapp S., Guabiraba R., Caballero I., Roesch F. (2022). New Insights in the Interplay Between African Swine Fever Virus and Innate Immunity and Its Impact on Viral Pathogenicity. Front. Microbiol..

[53] Thai M., Graham N.A., Braas D., Nehil M., Komisopoulou E., Kurdistani S.K., McCormick F., Graeber T.G., Christofk H.R. (2014). Adenovirus E4ORF1-induced MYC activation promotes host cell anabolic glucose metabolism and virus replication. Cell Metab..

[54] Thai M., Thaker S.K., Feng J., Du Y., Hu H., Ting W.T., Graeber T.G., Braas D., Christofk H.R. (2015). MYC-induced reprogramming of glutamine catabolism supports optimal virus replication. Nat. Commun..

[55] Heaton N.S., Randall G. (2010). Dengue virus-induced autophagy regulates lipid metabolism. Cell Host Microbe.

